# Dr. B. N. Colabawalla, FRCS, FAMS

**DOI:** 10.4103/0970-1591.42605

**Published:** 2008

**Authors:** S. S. Joshi

**Affiliations:** Department of Urology, Jaslok Hospital and Research Centre, Mumbai 400 006, India. E-mail: samjoshi38@hotmail.com

**Figure:**
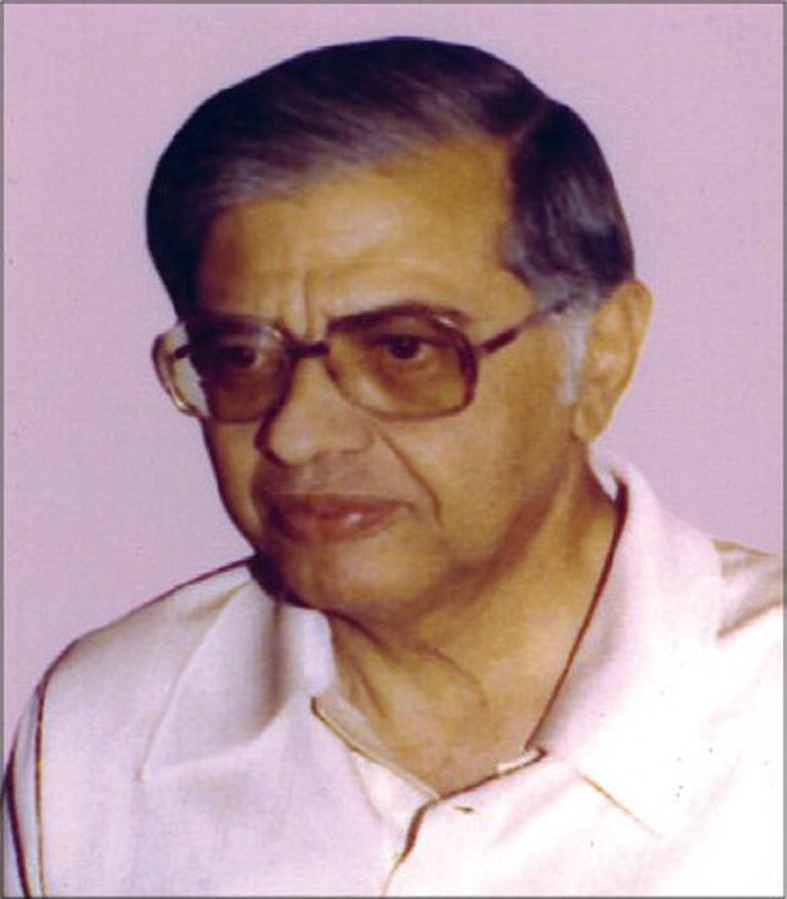
Dr. Colabawala

Dr. Burjorji Nusserwanji Colabawalla, popularly known as “Buji”, was born on 18 August, 1922 in a middlesclass Parsi family from Karachi and died after a long illness on 23 September 2002, in Mumbai.

Buji was studied at St. Patrick's High School, Karachi, and came to St. Xavier's College, Mumbai for further education. After completing his BSc, he joined the Grant Medical College in 1943. After completing his graduation, he proceeded to UK to do his postgraduate training in Surgery. He did his postgraduate training both at the Royal College of Surgeons, England and at Hammersmith Hospital under Prof. Ian Aird. He passed his FRCS (London) in 1954. Buji also worked in various hospitals as resident and was fortunate to work with Mr. Norman Tanner (a well-known gastroenterology surgeon of yesteryears) at the St. James Hospital, London. At this hospital he also worked with Mr. Henry Vernon and was introduced to the subject of urology as a specialty. This led to his working with one of the pioneers of British Urology – Mr. Hamilton-Stewart, at Bradford Royal Infirmary, as a registrar for two and a half years. He then returned to India in 1957.

On his return Buji decide to practice only “Urology” as a super-specialty. In 1957, it must have been indeed difficult to implement this decision with a growing family to support. Buji's wife Mehroo was a great supporter of Buji in this decision. (Mehroo was also able to handle volatile Buji with great ease, all she had to do was to up an octave and call him BAJYA instead of Buji!) Buji wrote in the Indian Journal of Surgery in 1966,[1] “Perhaps an important barrier is the psychological one of recognition as a specialty within the profession itself – an aspect that has confronted specialties in the past. If it is comprehended that the purpose of urology is not to supplant but merely to supplement surgical care of patients, then a major step in breaking down this barrier has been taken”.

Words of wisdom written in 1966! But how true they are even today, as urologists of India are now fighting to establish subspecialties in Urology and trying to end the “Turf war” which Buji described. Today's young Turks in urology need to thank pioneers like Buji for the freedom and monetary[1] gains in practicing only urology.

Buji established a separate department of Urology at St. George's Hospital, Mumbai and was joined by Dr. Anup Gokarn as consultant urologist. It soon established its credentials as an excellent unit to work for the knowledge and practice of urology. Unfortunately, Buji was never recognized as a teacher by the University of Mumbai, but his successor Dr. Anup Gokarn was recognized by the University as teacher after the university started its MCh course in urology. This mistake of the Mumbai university was corrected by other universities of India and departments of urology. He was visiting professor at the Christian Medical College, Vellore, SMS Medical College Jaipur and Kasturba Medical College, Manipal. As Prof. P Venugopal remarked to me “My first pyeloplasty as a resident in urology was assisted and taught by Buji and I still follow those principles”. He was a prominent faculty member of all continued medical education programs of the Urological Society of India, since its inception for many years. All of Buji's residents, and postgraduates of general surgery at the Grant Medical college benefited from his lectures and clinical discussions. But to get this benefit, one had to take Buji's sharp tongue for he did not tolerate fools and hypocrites. One also had to ignore his “second language”, for when he was irritated, like a true Parsi, this would flow fluently both in English and Parsi-Gujarati.

Buji was also a member of the expert committee for formulation of training programs for MCh in urology for the University of Madras and also subsequently an examiner. He was member of te editorial board of the British Journal of Urology. He was an expert advisor to the Government of India, Union Public Service Commission.

Buji was interested in research as well and was involved in two all-India trials – (1) Clinical and bacteriological study of genitourinary tuberculosis – ICMR project 1969-1973 as chief investigator, (2) Incidence of urolithiasis in India – ICMR technical report series no 8-1971.

Buji was the founder secretary-treasurer of the urology section of Association of Surgeons of India and became its President in 1971. He also delivered the inaugural Late Himadri Sarkar Memorial Oration on “Urinary calculus disease – perspective from history” in 1979. He was elected Fellow of the Academy of Medical Sciences of India in 1981.

In 1973 Buji's mentor Late Dr. Shantilal Mehta started Jaslok Hospital and Research Centre. He invited Buji to start a department of urology and renal transplantation (with Dr. M K Mani). The rest as the cliché goes is history. Jaslok Hospital now takes two students in urology per annum for a postgraduate course for the National Board of Examinations. It has an excellent record of success at Diplomate examinations. All postgrduates have settled well throughout India.

Buji spoke about this at every oppertunity be it at a Rotary meeting or a medical conference. This well seen by his being an important member of the constitutional committee of all three “avatars” of the Urological Society of India – viz.(1) urology section of Association of Surgeons of India (ASI), (2) Urological Society of India (under ASI) (3) Urology Society of India (separate body) with its four zones.

In 1970, Buji started with Kisan Mehta as the founder President, the National Kidney Foundation of India (NFK). This is a voluntary organization propagating free donation for therapeutical transplantation or anatomical research. Buji was soon disappointed with the commerce in renal transplantation. As the secretary of NFK and as a transplant surgeon of Jaslok Hospital, he wrote numerous letters to various authorities in Maharashtra and to the central Health Ministry. His efforts ultimately bore fruit when the Maharashtra transplantation Act was passed in 1982, but rules and regulation came much later. Buji spoke about this at every opportunity be it Rotary or a medical organization.

Ethics both in day to day life and medical practice were a great focal point for Buji. While speaking at a Seminar on “Brain death and organ transplantation” in Mumbai, and while giving the Dr. S K Pande memorial oration in Jodhpur, both in 1990, Buji said “It is postulated that an individual is free to donate his kidney for a price; hence why should the society object? The freedom of an individual to behave as he likes is circumscribed by the needs of the greater good of social morality! The replacement of the ethical concept of “intrinsic value” of humans by that of a concept of “extrinsic value” of the body or its parts makes it a saleable commodity with a price dictated by market forces”. Buji fought from the beginning till his death against “agents” for unrelated donors. That he did not succeed fully – apart from the transplantation Act, is seen in the exploits of two infamous agents from North and South of India.

One of the non-urological interests of Buji was his commitment to “Right to die with dignity”. Buji's mother had chronic kidney disease and he was just a hapless spectator of the sufferings. What bugged him was the negative answer to the question “Isn't there a humane way of ending her misery?” In 1980, Buji met Mr. Minoo Masani, Member of Parliament, and founder of the Society “Right to Die with Dignity” in Mumbai. Buji tirelessly propagated the Society's and his personal philosophy of a person's right to die with dignity that rested on the pillars of free will, freedom of choice, right of self-determination while maintaining the autonomy and dignity of individual. At the time of his death, Buji was the chairman of this Society. Buji was also deeply involved with the Rotary movement, and went on to become the President of Rotary Club of Bombay. His activities in Rotary were more concentrated on the Interact and Rotaract movements.

Buji was an atheist but had studied his own religion in detail as well as others like Hinduism, Islam etc. He and his wife were cremated as per their wishes and no funeral speeches were given. A voracious reader, his interest ranged from Bertrand Russell (his favorite author), to travelogues, books on music, urology etc. His other addiction was smoking. Buji without his beloved pipe was unthinkable! He was also fond of western classical music and over the years had collected an enviable collection. This is now with his daughter Khorshed, married and settled in the UK. Buji's wife Mehroo died suddenly due to a cerebrovascular accident. After her death, Buji who by then was not in good health due to Heaptitis C-induced cirrhosis, really lost interest in life and died in 2002. Buji's son Kershaw is married and lives in Toronto.
